# A peer-led learning program about intimate and romantic relationships for persons with mental disorders (AIRIKI): co-creation pilot feasibility study

**DOI:** 10.1186/s12888-023-05254-1

**Published:** 2023-10-19

**Authors:** Masako Kageyama, Keiko Yokoyama, Kayo Ichihashi, Shintaro Noma, Ryota Hashimoto, Misato Nishitani, Reiko Okamoto, Phyllis Solomon

**Affiliations:** 1 Osaka University Institute of Advanced Co-Creation Studies, 1-7 Yamadaoka, Suita, Osaka 565-0871 Japan; 2 Department of Nursing, Faculty of Nursing, Yokohama Soei University, 1Miho-cho, Yokohama, Kanagawa 226-0015 Japan; 3 Department of Neuropsychiatry, University of Tokyo Hospital, 7-3-1 Hongo, Bunkyo, Tokyo, 113-8655 Japan; 4 Peer support online group ‘Hamaccha’, Yokohama, Japan; 5 Department of Pathology of Mental Diseases, National Institute of Mental Health, National Center of Neurology and Psychiatry, 4-1-1 Ogawahigashi, Kodaira, Tokyo, 113-8655 Japan; 6 Department of Health Promotion Science, Osaka University Graduate School of Medicine, 1-7 Yamadaoka, Suita, Osaka 565-0871 Japan; 7 School of Social Policy & Practice, University of Pennsylvania, 3701 Locust Walk, Philadelphia, PA 19104-6214 USA

**Keywords:** Mental disorders, Romantic relationships, Personal recovery, Peer support, Co-creation, Intervention

## Abstract

**Background:**

Intimate and romantic relationships are important in life for individuals, irrespective of mental health status. We developed a four-hour peer-led learning program for persons with mental disorders about intimate and romantic relationships through a co-creation process with service users and examined its preliminary effectiveness and feasibility of implementing the program.

**Methods:**

A one-group pretest–posttest trial was conducted using a mixed-method design for 45 individuals with mental disorders in Japan. Outcome data were collected at three time points: baseline, post-intervention, and one month after program completion. Mixed models for repeated measures (MMRM) were used to examine changes over time in the Rosenberg Self-Esteem Scale (RSES), Recovery Assessment Scale (RAS), Herth Hope Index (HHI), and the original items. Group interviews were conducted for process evaluation.

**Results:**

MMRM showed significant changes over time on RSES, RAS, HHI, and two original items “I am able to communicate well with others about myself” and “I am able to listen to others well.” In multiple comparisons, RSES and HHI were significant one month after the program. Participants reported changes during the first month after attending the program in terms of their positive attitude toward romantic relationships (n = 14), taking romantic actions (n = 11), and feeling their overall communication improved (n = 11). Although two participants had an unscheduled psychiatric visit that could be attributed to attending the program, all recovered after one month.

**Conclusions:**

The program exhibited preliminary effectiveness to a moderate extent in improving recovery, particularly regarding self-esteem and hope. The program is feasible but requires further modifications regarding inclusion criteria for participants and the training of peer facilitators.

**Trial registration:**

UMIN000041743;09/09/2020.

**Supplementary Information:**

The online version contains supplementary material available at 10.1186/s12888-023-05254-1.

## Background

The concept of *recovery* has become mainstream in mental health services in Western countries [1]. Personal recovery at its core is an individual process of learning how to live well and how to get along with or without enduring symptoms or vulnerabilities [2]. Systematic reviews focusing on personal recovery have shown that social connections are an important component of recovery [3–5]. Among social connections, intimate and romantic relationships may be viewed as basic personal expressions of humanity that influence meaningful living [6]. According to the 2018 Japanese National Character Survey, “family” is the most important phenomenon that Japanese value, followed by “love” [7]. Intimate and romantic relationships are important in life for Japanese individuals, irrespective of mental health status. Moos and Schwebel defined intimacy in enduring romantic relationships as determined by the level of commitment and positive affective, cognitive, and physical closeness one experiences with a partner in a reciprocal relationship [8]. Such intimate and romantic relationships have received limited attention in mental health services and recovery research [9, 10].

In a systematic review of 20 studies of persons who experienced psychosis, 15% of participants were married [9], which is considerably lower than almost half of the general population in the UK [11] and the U.S.A. [12]. The situation in Japan is similar, where 15–27% of people with mental disabilities are married [13–15], which is lower than the 55.6% of the total population [16]. A Japanese survey showed that about 70% of people with mental disorders who did not have a romantic partner wished to have one [14].

For persons with psychosis, symptoms such as anxiety, depression, cognitive problems, and fatigue due to mental exhaustion can be barriers to initiating and engaging in romantic relationships, given the tendency by individuals experiencing such symptoms to isolate themselves [6]. With prolonged social isolation, some people who experience psychosis and serious mental illness (SMI) feel unable to engage in and manage conversations [6, 17]. In a qualitative study for persons with schizophrenia, they perceived difficulties in understanding others’ intentions, along with the lowering of trust in themselves and others as potential psychological barriers [18]. In addition, sexual dysfunction due to side effects of medications can act as a barrier [19]. Individuals who have experienced psychosis and SMI tend to have self-stigma, a loss of confidence, low self-esteem [17, 20], and a fear of being rejected upon disclosing their illness [19]. For persons with psychosis, a lack of financial resources can function as another barrier [18, 21]. A systematic review of marital relationships among people with bipolar disorder reported a high divorce rate owing to the burden of spousal care, self-sacrifice, and other negative effects associated with the condition [22]. Another systematic review of quantitative studies [21] reported that persons with psychotic disorders face numerous barriers, such as those discussed above, to forming and maintaining intimate and romantic relationships.

Through a qualitative systematic review of the literature on experiences and support needs of persons with SMI regarding sexuality and intimacy, the investigators concluded that those with SMI possess the will and desire for intimate and romantic relationships despite potential barriers [17]. Therefore, support is needed to help them overcome barriers to intimate and romantic relationships. A review of qualitative research on romantic and sexual relationships found a gap between the professionally assessed psychiatric needs of people with mental disorders and the needs expressed by those with the disorders, concluding that future research should focus on psychosocial approaches to address the unmet needs articulated by persons with mental disorders [19]. Training programs providing support and education regarding intimate and romantic relationships are essential to meet these desires for people with mental disorders and to facilitate their recovery [23]. Social skills training has long been available as an intervention program for romantic relationships [24], and a pilot study of seven participants in a cognitive-behavioral therapy group program named “The Power of Two” has recently been reported [25]. However, evaluations of intervention programs for intimate and romantic relationships are considerably less frequently reported than those of sexual health intervention programs [26, 27]. Intervention programs for intimate and romantic relationships are expected to enhance one’s self-esteem toward developing such relationships [6], a primary element of recovery that plays a central role in forming these relationships. Furthermore, for many Japanese who place the highest value on family and love [7], not giving up on forming intimate and romantic relationships is thought to give them hope for the future.

Peer support is crucial for many people with mental disorders during the recovery process [2]. Systematic reviews of peer-led support programs have reported that while they are not superior to programs offered by professionals, they are neither inferior nor harmful [28, 29]. Although mental health services have traditionally been developed by health professionals, the concept of co-production with service users has recently been identified as central to the provision of recovery-oriented services [1]. Service users’ experiences should be a major component in the design and delivery of recovery education [30]. However, the concept of co-production is relatively new, with some ambiguities in its definition [1]. Pearce et al. delineated aspects of a similar concept of co-creation, including co-production. The co-creation of new knowledge involves four collaborative processes: co-ideation, co-design, co-implementation, and co-evaluation [31]. Consequently, this study aimed to develop a peer-led learning program about intimate and romantic relationships through co-creation with service users and to examine the preliminary effectiveness and feasibility of implementing the program.

### Process of program development

The program was developed with peers with mental disorders (peer collaborators) involved in all four processes in the co-creation of new knowledge [31].

The first process is “co-ideation,” which involves engaging in open dialogue to share new and creative ideas for solving problems related to new programs [31]. In this study, peer collaborators interested in intimate and romantic relationships were recruited through a large self-help group that had previously hosted events concerning the intimate and romantic relationships of persons with various mental disorders. Two researchers (MK and KY) — both of whom are mental health nurses — conducted seven meetings over 14 h in 2018 with 16 peer collaborators and one partner, sharing their experiences regarding intimate and romantic relationships. Researchers and peer collaborators organized their experiences into barriers and solutions to intimate and romantic relationships for persons with mental disorders. These experiences were later published in a book [32].

Second, the process of “co-design” developed a description of the technical details of the new program (prototype), as well as the research design to be used to evaluate the program (protocol) [31]. We held 12 meetings, totaling 49 h, with nine peer collaborators who, among those who participated in the co-ideation process, were willing to participate as co-designers. Peer collaborators proposed various ideas for program goals, style of implementation, substantive content, role-plays, and so on. The program was first developed using input from peer collaborators. Findings from the extant literature were then discussed, and information regarding sexually transmitted diseases, unwanted pregnancy, and domestic violence was added as elements for risk reduction [19, 21]. The program was modified numerous times during the peer collaborators’ implementation of the prototype program. During implementation, it was determined that role-playing to improve social skills would likely be effective. Consequently, two role-play scenarios were included in the program. Two psychiatrists (KI and RH) became involved in the program when it was near completion and reviewed the program’s content and protocols from a medical perspective. The intervention study’s potential participants criteria, recruitment methods, and effectiveness outcomes were all reviewed with these peer collaborators as part of finalizing procedural decisions.

The third process of “co-implementation” ensured that the co-designed program was in accordance with the research protocol [31]. Four peer collaborators who continued from the co-production process and the researchers played a central role in the co-implementation. Five facilitator training workshops were publicized and held. Fifteen facilitators were trained, including four peer collaborators, who then facilitated the 8 program sites.

The final process of “co-evaluation” embeds data collection or other formal research techniques into the co-implementation process and jointly interprets the meaning and implications of the results [31]. After the intervention study was completed, we held three meetings with the peer collaborators to discuss minor improvements and dissemination of the program.

## Methods

### Design

A one-group pretest–posttest trial was conducted using a mixed-method design. Participants were assessed for outcomes using self-administered questionnaires at baseline (T1), post-intervention (T2; the day after T1), and one month after program completion (T3). The paper questionnaires were distributed and collected by the principal researcher. Group interviews (with some individual to accommodate schedules) were conducted by the principal researcher at T3 for process evaluation (time span per person was 12–21 min). The intervention and data collection were conducted between March and December 2022. This project was registered in the Clinical Trial Registry (UMIN 000041743; 09/09/2020).

### Program description

The program was named AIRIKI (“the power to love” in Japanese). This program offers essential elements for overcoming barriers related to forming and maintaining of intimate and romantic relationships among those with mental disorders. The program does not aim to provide knowledge per se but to enhance participants’ own awareness and develop their own ideas about relationships by considering those with their own loved ones through sharing their experiences and thoughts. This structured program is conducted face-to-face in small groups of at most 10 people and promotes peer-led learning in which both facilitators and participants are persons with mental disorders. The program provides detailed instructions, including principles such as respect for different values, including sexual diversity, and notes the number of minutes to be allotted to each component. With reference to a family peer learning program [33, 34], the core structure of this program involves teaching strategies that combine the sharing of experiences through the use of a simple guidebook and peer group facilitation using group work skills to provide positive feedback [34]. The current program was conducted over two days with four modules of 60 min each, using an originally developed simple guidebook.

Table 1 lists the components of the program. In Module 1, participants deepen their thinking about love and romance with reference to theories of love [35, 36], and connect love and romance with that of their own lives. In Module 2, the participants consider what is important in intimate and romantic relationships with their partners — in reference to Fromm’s theory of love [36]. Moreover, a role-playing exercise is included to facilitate communication with others in a respectful manner. In Module 3, participants learn how to find a partner and dating etiquette. Module 3 includes how to communicate about one’s illness. In Module 4, the participants share their thoughts and experiences regarding marriage. Finally, the participants share their strengths and messages of support. Program participants subsequently describe how they would like to act in the future. We hypothesized that AIRIKI would increase participants’ self-esteem, sense of hope, and recovery at termination of the program and one-month after program completion.

**Table 1:** Components of the program

				Structure
				Program focused on romantic relationships and love
				Small group of less than 10 people, including only those with mental disabilities
				Groups led by peer facilitators
				Sharing own thoughts and experiences based on brief explanations
Modules	min	Lesson	Purposes	Contents
0-Warming-up	20		Relieve tension, knowing each other	Self-introduction, motivation for participation, type of persons you like, how you feel today
1-What is love for you?	8	Introduction	Guarantee safe and secure place	Goals and ground rules of program
	7	Image of love	Have person’s share thoughts about love and romance	Text briefly introduces theories about love and romantic relationships, and participants share their thoughts and experiences. They identify love not only from their partners but also from their parents and others.
	10	Love and romance		
	5	Subjects to love		
	20	Important persons and events for your life	Confirm love of those who have supported participants	Participants list people and events that are important in their lives and recovery and share them with other participants.
	10	Reflection	Clarify own thoughts	Participants share their insights and impressions.
2-Understand your loved one	20	What it takes to love others	Clarify what is important to love others	Based on Erich Fromm’s theory, care, responsibility, respect, and knowing are introduced briefly as common elements of love. Personal boundaries are briefly described. Participants share their thoughts and experiences.
	10	Basic communication skills	Learn communication skills	Basic communication skills such as listening and ‘i message’ are explained. Participants share what they try to do in their communication.
	20	Skills training for gift giving		As an exercise in communication, participants role play with each other in a situation where a birthday gift is given to a partner.
	10	Reflection	Clarify own thoughts	Participants share their insights and impressions.
3-Meeting and dating	5	Warming-up	Relieve tension and guarantee a safe place	Beginning of second day; Facilitators assess mood of the day and state ground rules of program.
	10	Finding a partner	Know own values regarding love	Participants share what they value when choosing a partner and discuss about how to find a partner.
	8	Dating etiquette	Facilitate relationships with a partner	Participants share what they are aware of as dating etiquette.
	27	Skill training in communicating illness	Learn ways to communicate about illness with a partner	Important to know how to tell your partner about mental illness. Participants consider whether or not to disclose their illness to their partner and practice in their own way.
	10	Reflection	Clarify own thoughts	Participants share their insights and impressions.
4-Retaining a long-term relationship	5	What to watch out for in a relationship	Avoid risks in romantic relationships	Mental health conditions, sexually transmitted diseases, unwanted pregnancies, and domestic violence are briefly explained.
	10	Marriage	Consider whether to get married or not and think about solutions to problems in marriage	Different forms of long-term relationships including marriage and cohabitation and what to share with your partner in marriage are explained. The side effects of medications on sexual difficulties and child care are also explained. Participants interrogate married participants and facilitators and share their concerns and coping strategies with each other.
	40	Reflection and positive feedback	Remind what participants learned, be aware of their own strengths	Participants present what impressed them about program and own future plans. All participants and facilitators share their strengths and messages of support with each other. Finally, participants presented how they would like to act in the future.
	5	Overall comments	Program completion	Participants share their overall thoughts of entire process.

### Study participants

Participants comprised adults (20 years of age or older) who met all of the following criteria: (1) diagnosed with a mental disorder and currently undergoing psychiatric outpatient treatment; (2) psychiatrist’s determination that the patients’ psychiatric symptoms were stable enough to allow participation in the study; (3) psychiatrist’s determination that participation in this study was not expected to interfere with psychiatric treatment; (4) able to communicate well enough to understand the explanation of the study and to make a decision on their own regarding study cooperation; and (5) able to participate in group discussion activities without difficulty using Japanese.

The sample size was determined based on the effect size obtained from the results of a pilot randomized controlled trial (RCT) conducted prior to this study. An effect size of 0.5, α = 0.05, and β = 0.20, revealed the required sample size to be 33. The sample size was set to 40, considering dropouts. As the program was implemented on a group basis, the program implementation was planned and participants were recruited until more than 40 were enrolled. The final sample size was 45.

### Program implementation

Information on program implementation was widely publicized through the program’s website, e-mail lists, social networking services of several mental health self-help groups, and flyer distribution. Once we received a call from a potential participant, we planned an intervention near where the person lived, and distributed flyers in that area to encourage further participation with a reward ($23). The program was implemented face-to-face at eight sites across prefectures, thus covering a wide area in Japan. Once an application was received, the researcher sent a research description (instructions for the psychiatrist were enclosed) and the researcher explained the study over the phone. The applicant then gave the research description to his/her psychiatrist, who wrote a decision on whether or not he/she was approved to participate, subsequently sending it to the researcher by mail. The information regarding research participation was explained again on the day of the start of the program, and written informed consent was obtained from all participants.

### Outcome measures

To measure the program’s objective of promoting recovery, the Rosenberg Self-Esteem Scale (RSES), Recovery Assessment Scale (RAS), and Herth Hope Index (HHI) were used as outcome measures. The RSES and HHI measure the major components of the concept of recovery [3].

#### Rosenberg self-esteem scale (RSES)

The RSES is a self-report scale that measures self-esteem. The validity and reliability of the Japanese version have been confirmed [37, 38]. The RSES consists of 10 items rated using a four-point Likert scale ranging from 1 (strongly disagree) to 4 (strongly agree). The total score ranged from 10 to 40 points, with higher scores indicating higher self-esteem. Cronbach’s alpha coefficients in this study ranged from 0.757 to 0.888 across the three time points.

#### Recovery assessment scale (RAS)

The RAS is a self-report scale that measures the recovery process of persons with mental disorders using a five-point Likert scale ranging from 1 (strongly disagree) to 5 (strongly agree). Higher total scores indicate being further along in the process of recovery, and the reliability and validity of the 24-item Japanese version of the RAS have been confirmed [39]. In this study, the mean score was calculated. Cronbach’s alpha coefficient in this study was 0.922–0.952 across the three time points.

#### Herth hope index (HHI)

The HHI is a self-report scale that measures levels of hope. The validity and reliability of the Japanese version have been confirmed [40, 41]. The HHI consists of 12 items rated on a four-point Likert scale ranging from 1 (completely disagree) to 4 (completely agree). The total score ranged from 12 to 48 points, with higher scores indicating higher levels of hope. Cronbach’s alpha coefficient in this study ranged from 0.863 to 0.918 across the three time points.

### Process evaluation measures

Process evaluation was conducted using originally developed items and interviews. We developed eleven items on relationships with significant others that were relevant to program content with input from peer collaborators. Peers felt it was important to determine if there were any changes in these process dimensions. For the following items, respondents were asked to choose the number that best fit their feelings and thoughts from not at all true (1) to totally true (7). The items were “I want to understand myself,” “I want to understand others,” ”I want to take care of myself,” “I want to take care of my friends,” “I want to take care of my partner,” “I want to take care of my family,” “ I am able to love others,” “I am able to appreciate the values of others,” “I am able to see the good side of things,” “I am able to communicate well with others about myself,” and “I am able to listen to others well.”

The interview-based process evaluation was based on an in-person interview obtained at T3. One month after participating in the program (T3), participants were asked what impressed them and how they changed after participating in the program.

### Feasibility evaluation measures

Feasibility was determined by subjective ratings of satisfaction with the program and whether they thought it would help them in their lives, as well as by any negative effects of the program. Specifically, unscheduled visits to the psychiatrist up to one month after completion of the program (T3 questionnaire), the number and reasons for those who dropped out of the program, and deterioration of mental health condition after completion of the program (T3 interview) were assessed. For those who dropped out, they were contacted on the second day of the program and a few days later to check on their mental health status.

### Analysis

The dataset included all participants who provided data at T1. Baseline characteristics are summarized as means and standard deviations for continuous variables and frequencies and proportions for categorical variables. For the outcome measures and process evaluation measures, we first compared the means of the three time points. Because this program was implemented on a group basis, data is nested by group. Therefore, we fitted mixed models for repeated measures (MMRM), with time points (T1, T2, and T3) as fixed effects and program sites as random effects. If the outcome measures showed significant changes in MMRM over time, T1–T2 and T1–T3 multiple comparison tests (Bonferroni correction) and effect sizes (Cohen’s d) were performed to examine the differences between time points. A *p*-value of 0.05 or less was treated as significant. Cohen’s d coefficients were considered correlation coefficients [42] and were calculated using G*Power. All other analyses were performed using SAS 9.4 (SAS Institute Inc., Cary, NC, USA).

The interviews were recorded and converted into textual data. The textual data were summarized for each statement made by the participant. The participants were classified according to their study IDs. The number of participants who reported about what impressed them regarding the program was counted based on the summarized statements for each program’s structural elements and content themes. The data on changes after program participation were categorized based on the similarity of the summarized statements, and the corresponding number of participants was counted. A principal researcher established the program’s structural elements, content themes, and categories. Counts were conducted separately by two researchers (MK and MN) for purposes of reliability (rate of agreement of 94.4%), and areas where there were differences in classification were reviewed by a third researcher (RO) to decide on the category.

## Results

### Study participants

**Fig. 1:**
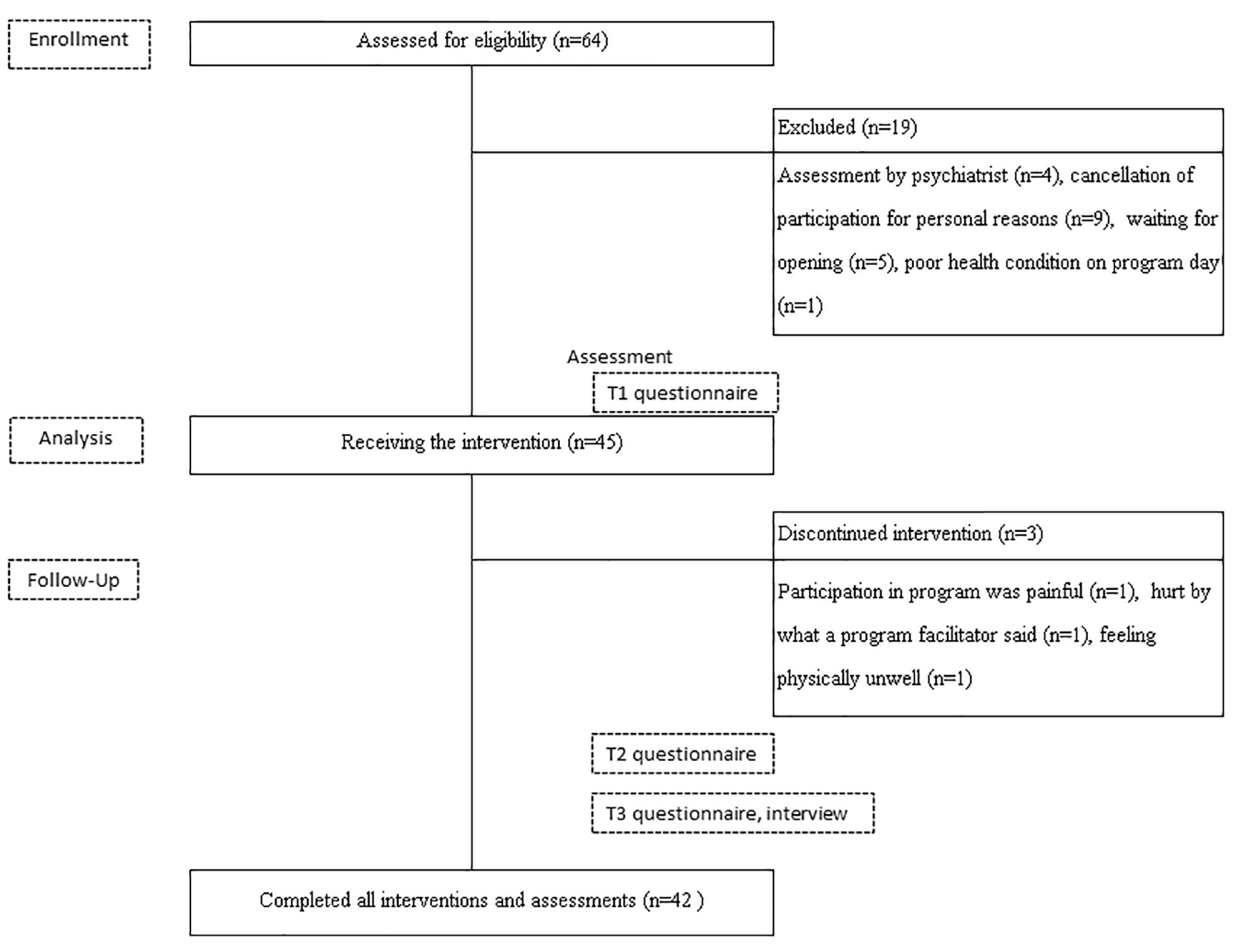
Flow of study participants

Figure 1 depicts the flow of study participants. Sixty-four persons applied to participate, but four did not meet the inclusion criteria because their psychiatrist determined that their psychiatric symptoms were not stable enough for participation in this study. Of the applicants, nine withdrew for personal reasons, five were unable to participate because they were on the waiting list for the program site and the date they wanted, and one was unable to participate because of poor health. Forty-five participants completed the baseline questionnaire (T1) and participated in the program. However, three completed Modules 1 and 2 on the first day, but did not show up on the second day, thus dropping out. Finally, 42 participants completed the entire program, including all assessments and interviews at one month follow up.

### Baseline characteristics of study participants

Table 2 presents the participants’ demographic characteristics. The participants were comprised of 26 males (57.8%) and 19 females (42.2%) (self-reported), with an average age of 40 years (range: 24–58). In terms of residential status, 18 participants (40.0%) lived alone and 17 (37.8%) lived with their parent(s). Regarding marriage, 32 participants (71.1%) had never been married, while 33 participants (73.3%) had a previous partner and 14 (31.1%) had a current partner. The primary psychiatric diagnoses reported by the participants were schizophrenia in 16 (35.6%), neurodevelopmental disorders (autism spectrum disorders and/or attention-deficit/hyperactivity disorders) in 14 (31.1%), mood disorders in 11 (24.4%), and anxiety disorders in 4 (8.9%). Two participants (4.4%) had mild intellectual disability in addition to their psychiatric disorder. Excluding developmental and intellectual disabilities, the average age at onset of mental disorders was 24.6 years (range: 11–43). All but one had regular psychiatric visits, and all but four used public services for their mental disability. However, nearly half (21 participants) were working without support services. The motivations for participation were as follows: 24 (53.3%) were interested in romance, and 19 (42.2%) wanted to be in a romantic relationship or wanted to think about how to improve their relationship with their partner.

**Table 2:** Demographic data of participants

		N=45
Items		n (%), Mean (SD)
Gender (self-reported)	Male	26 (57.8%)
	Female	19 (42.2%)
Age	Mean (SD)	40.0 (8.5)
Residential status	Living alone	18 (40.0%)
	Living with parent(s)	17 (37.8%)
	Living with partner	7 (15.6%)
	Living only with child(ren)	1 (2.2%)
	Living with support	2 (4.4%)
Marriage status	During marriage	7 (15.6%)
(Not mutually exclusive)	Experience divorce	7 (15.6%)
	Unmarried	32 (71.1%)
Previous partner(s)	Yes	33 (73.3%)
	No	12 (26.7%)
Current partner	Yes	14 (31.1%)
	No	31 (68.9%)
Primary psychiatric diagnosis	Schizophrenia	16 (35.6%)
	ASD and/or ADHD	14 (31.1%)
	Mood disorders	11 (24.4%)
	Anxiety disorders	4 (8.9%)
Complication	Mild intellectual disability	2 (4.4%)
Age at onset of illness*	Mean (SD)	24.6 (8.4)
Psychiatrists	Regularly visited	44 (97.8%)
	Not regularly visited	1 (2.2%)
Psychiatric hospitalization	None	18 (40.0%)
	Experienced	27 (60.0%)
Disability services	Disability pension	29 (64.4%)
(Not mutually exclusive)	Disability certificate	39 (86.7%)
	Neither	4 (8.9%)
Social participation	Stay at home	5 (11.1%)
	Using rehabilitation services	11 (24.4%)
	Using employment support	7 (15.6%)
	Employment without support services	21 (46.7%)
	School attendance	1 (2.2%)
Motivation to participate	Interested in romance	24 (53.3%)
	Want to be in a romantic relationship	19 (42.2%)
	Want to think about how to improve my relationship with my partner	19 (42.2%)
	Others	23 (51.1%)

ASD: Autism Spectrum Disorder

ADHD: Attention-Deficit/Hyperactivity Disorder

* Excluding congenital disabilities (ASD and/or ADHD and mild intellectual disabilities)

### Quantitative analyses

#### Effectiveness over time

As shown in Table 3, the MMRM results revealed significant changes over time in RSES (F = 4.44, p = 0.014), RAS (F = 3.96, p = 0.022), and HHI (F = 7.71, p < 0.001). Multiple comparison tests (Bonferroni correction) for RSES and HHI remained significantly higher than baseline (T1), not only immediately after attending the program (T2), but also one month after attending the program (T3). These effect sizes (Cohen’s d) varied between 0.383 and 0.460, slightly lower than the moderate 0.5. However, RAS were significantly higher immediately after attending the program (T2) than at baseline (T1), but were no longer significantly different one month after program termination (T3).

**Table 3:** Outcome and process evaluation measures

	Baseline (T1) (n = 45)		Post (T2) (n = 42)		Endpoint (T3) (n = 42)		MMRM (T1-T2-T3)		Paired t-test (adjusted p)		Correlation (r)		Cohen’s d (d)	
	Mean	SD	Mean	SD	Mean	SD	F	p	T1-T2	T1-T3	T1-T2	T1-T3	T1-T2	T1-T3
Rosenberg Self-Esteem Scale	22.76	4.30	24.40	5.09	24.26	5.87	4.44	0.014*	0.023*	0.019*	0.590	0.755	0.383	0.391
Recovery Assessment Scale	3.38	0.65	3.59	0.64	3.50	0.70	3.96	0.022*	0.015*	0.221	0.678	0.658	0.405	0.214
Herth Hope Index	31.87	6.24	33.86	6.31	33.29	6.74	7.71	< 0.001*	0.002*	0.005*	0.762	0.853	0.460	0.400
Original developed items for process evaluation														
I want to understand myself	6.11	1.27	5.86	1.49	6.05	1.23	0.88	0.417						
I want to understand others	5.93	1.01	5.93	1.45	6.07	1.07	0.52	0.598						
I want to take care of myself	5.91	1.26	5.83	1.38	5.67	1.57	0.65	0.522						
I want to take care of my friends	6.29	0.87	6.26	1.06	6.31	0.95	0.06	0.939						
I want to take care of my partner	6.36	0.88	6.29	1.20	6.29	1.20	0.10	0.907						
I want to take care of my family	6.16	1.04	6.12	1.13	6.00	1.33	0.79	0.457						
I am able to love others	5.00	1.67	5.24	1.45	5.05	1.55	1.50	0.228						
I am able to appreciate the values of others	5.47	1.41	5.38	1.40	5.29	1.37	0.41	0.664						
I am able to see the good side of things	5.13	1.46	5.40	1.29	5.00	1.67	2.42	0.093						
I am able to communicate well with others about myself	3.67	1.61	4.24	1.68	4.10	1.68	3.21	0.044*						
I am able to listen to others well	4.24	1.45	4.79	1.26	4.74	1.47	5.03	0.008*						

MMRM: A mixed model for repeated measures with time points (T1, T2, and T3) as fixed effects and program sites as random effects

Paired t-test: multiple comparison test using Bonferroni correction

Cohen’s d coefficients were calculated considering correlation coefficient

*: p < 0.05

#### Process evaluation

As shown in Table 3, MMRM for process evaluation measures revealed significant changes over time in the following items: “I am able to communicate well with others about myself” (F = 3.21, p = 0.044) and “I am able to listen to others well” (F = 5.03, p = 0.008). No significant changes were observed over time for the other originally developed items.

#### Feasibility evaluation

Regarding program satisfaction, 29 (69.0%) were “very satisfied,“ 12 (28.6%) were “fairly satisfied,“ and 1 (2.4%) was “not very satisfied”; notably, 0 (0.0%) reported “not satisfied at all.” As for whether they thought it would be useful in their lives, 29 (69.0%) thought it would be “very useful,“ 11 (26.2%) thought it would be “fairly useful,“ and 2 (4.8%) thought it would be “not very useful”; no participant 0 (0.0%) thought it would be “not useful at all.”

### Qualitative analyses

#### Process evaluation

Interview data from one month after program participation were analyzed to determine what was impressive about the program (Table 4) and the changes after program participation (Table 5).

**Table 4:** Positively impressive components of program

Structure		n	Narrative examples
Sharing own thoughts and experiences		25	I had a wonderful time listening to people share various values and real-life experiences, and I was able to speak up as well.
Small group of only people with mental disorders		17	It was a place to talk with people who have the same mental disorders as me, so I talked about things that I couldn’t easily discuss.
Role-playings		10	I’m glad I actually did it through role-playing instead of just talking.
Groups led by peer facilitators		8	Some of the facilitators were actually married, so I thought that maybe I could be with a partner like them too.
Focused on romantic relationships and love		8	I have never really talked to anyone about romantic relationships before, so I am really glad that I was able to talk about it.
**Modules**	**Lesson**	**n**	**Narrative examples**
1-What is love for you?	Image of love	1	I remember the kindness and compassion we had when we first started dating.
	Love and romance	9	It was also very important for me to clearly realize what I valued very much through what was written in the text.
	Subjects to love	5	I realized that I had been properly loved and nurtured as a child, so I thanked my mother.
	Important persons and events for your life	3	Hearing about a real-life experience of recovery as a result of love, and also remembering my own experience of being saved by love, I realized again that love is an important thing in life.
2-Understand significant others	What it takes to love others	5	I thought there was a difference between dependence and love.
	Communication skills, role-play for gift giving	9	Through role-playing, I learned that it’s not only about what I like, but I also have to figure out what my partner likes and match it to him or her.
3-Meeting and dating	Finding a partner	0	
	Dating etiquette	2	It was a rediscovery that without minimum manners like business etiquette, romantic relationships would not develop.
	Role-play in communicating illness	6	I had never been able to communicate my illness to others, but I realized that I could, depending on how I communicated.
4-Retain a long-term relationship	What to watch out for in a relationship	2	I realized that romantic relationships do not always lead to recovery, and that there can be negative aspects.
	Marriage	2	I realized that marriage is not only about living together, as I learned that there are also separate marriages and weekend marriages.
	Reflection and positive feedback	6	I received encouragement from others. In the future, when I have a hard time in my life, I will remember this encouragement and try to do my best again.

**Table 5:** Qualitative findings of changes after program

Categories	n	Narrative examples
Positive changes after program		
More positive attitude about romantic relationships	14	I had only been thinking about romantic relationships but not acting on it, but after hearing the facilitator’s words in the program, I was inspired to take action
Taken romantic actions	11	I felt more positive and confessed my feelings to the person I liked.
Improved communication generally	11	I have learned to listen to people through the program, which has helped me work on my customer service skills, as I used to have a hard time with it.
Acted to take better care of their families	4	I realized that it was because of my mother that I was able to love others, so I called her and said, “Thank you.“
Increased self-esteem	4	Communicating with others has increased my appreciation of myself.
Improved communication about romantic relationships	3	I am now more willing to talk to my psychiatrist and support staff about marriage and relationships.
Emotions of love arise	2	My feelings of love had stopped, but now my emotions are starting to grow again.
Acted to take better care of themselves	2	To take care of myself, I cleaned the bathroom first.
Made new friends	2	I made friends with the other participants and that was my change.
Others	5	
Negative changes after program		
Unstable mental health condition	4	Because my situation was different from that of the program participants, I was unable to be honest about myself and my participation in the program was stressful.
Less self-confidence	2	I lost confidence for a week because other participants were communicating better than me.
No change after program		
No change happened	3	No changes have been observed in particular yet; I’m still looking for a romantic relationship but it takes me a long time to act.

As shown in Table 4, things that positively impressed them about participating in the program included sharing thoughts and experiences (n = 25) with a small group of people with the same disabilities (n = 17), role-playings (n = 10), being led by peer facilitators (n = 8), and dealing with the theme of love (n = 8). These were related to the structure of the program. As described in the narrative examples, they said that sharing their thoughts and experiences focusing on romantic relationships and love broadened their ideas, and meeting peer facilitators who were married gave them hope for their own possibility of marriage.

In Module 1, the most common response was the focus on “love and romance” (n = 9) left the greatest impression on participants, and it helped them clarify their own ideas by sharing their thoughts with others. The next most common response was “subjects to love” (n = 5), in which the theme of parent-child love in addition to heterosexual love was discussed, with some participants confirming that they had received love from their parents. In Module 2, the most impressive part was the communication skills and the role-playing exercise of gift-giving (n = 9). They indicated that it helped develop the skills of communicating in a respectful manner regarding their partner’s preferences and feelings. In Module 3, the most impressive part was the role-playing exercise regarding communicating about their illness (n = 6). They learned there were various ways to articulate their illness to others for it to be understood. In Module 4, the most impressive part was reflection and positive feedback (n = 6). The positive feedback provided by others helped participants gain their own confidence.

As shown in Table 5, the most common change after participating in the program was a more positive attitude about romantic relationships (n = 14), followed by those who engaged in romantic actions to get closer to their partner (n = 11), and feeling that their communication in general had improved (n = 11). Others said their actions regarding care for their families had improved (n = 4), and their self-esteem had increased (n = 4). Some people spoke of negative changes after the program (n = 6), while others spoke of no changes (n = 3).

#### Feasibility evaluation

Nine participants reported negative changes in their condition and/or feelings during and up to one month after participation in the program [Additional File 1]. We found that two participants had unscheduled psychiatric visits after the program: one due to feeling stressed during the program because her situation was very different from those of other participants. The other reason was that the person enjoyed the program, but her mood was higher than normal. They both participated in the interview one month after program completion and were in good mental health condition at that time. Three reasons for dropping out were that one remembered painful experiences from listening to others, one was hurt by something unpleasant said by a program facilitator, and one became physically ill. Two participants, although they did not have an unscheduled psychiatric visit, indicated that their mental health condition had worsened. One had a medical condition that was exacerbated by the season, and one felt stressed due to becoming more aware of their own lack of a love life and felt unable to overcome the challenges of changing the situation. Two participants said they had lost confidence in themselves when comparing themselves to other participants. One said he lost confidence because others were better communicators than he was, and the other said she lost confidence because others were doing well in their marriage. As a result, of these nine participants who reported negative changes, seven experienced heightened psychological emotions due to the program. Although some adverse events occurred due to program participation, no serious adverse events were observed.

## Discussion

We developed a peer-led learning program on intimate and romantic relationships aimed at promoting recovery in persons with mental disorders. This section discusses implications of the preliminary effectiveness and feasibility of the program.

### Study participants

Although the study participants had a variety of primary illnesses, all but four were using disability services, indicating that most had some disability in their daily and social lives. However, 21 (46.7%) were working without support and 18 (40.0%) were living alone. According to a national survey of people with disabilities in Japan, 18.6% of people with mental disabilities under the age of 65 live alone, and 49.8% spend most of their daytime hours at home [15]. Thus, the participants in this study were in good condition when compared to all persons with disabilities, with many becoming more independent in their employment and living arrangements. Individuals at this stage are more likely to benefit from participating in this program.

### Effectiveness of the program

This program was found to be associated with significant changes over time in self-esteem, comprehensive recovery, and a sense of hope; however, not all had high effect sizes. Comprehensive recovery showed significant effects after immediately attending the program; however, self-esteem and sense of hope were significant up to one month after the program. Therefore, the program was preliminarily effective to a moderate extent in improving recovery, especially in terms of self-esteem and hope.

Participants also reported changes in their positive attitude toward romantic relationships (n = 14) and engaged in actions to further their romantic relationships (n = 11). In a previous study [6], it was reported that when self-esteem increases, persons with mental disorders are more able to express their thoughts and feelings to their loved one. In addition, the presentation of the action plan at the end of the program made it easier for the participants to take action after the program. The program was also memorable as they connected with others who shared their same disabilities, were given hope by meeting with a married peer facilitator, and were encouraged by the positive feedback they received from others. These findings are consistent with a study that found that seeing successful peers is inspiring and hopeful [43]. These elements overlap with the five processes of recovery: connectedness, hope, and optimism about the future, identity, meaning in life, and empowerment [3]. The RAS is a comprehensive measure of recovery [39] that includes some items closely aligned with the program, such as goal/success orientation and hope and personal confidence, as well as others not intended to be addressed by the program, such as reliance on others, lack of domination by symptoms, and willingness to ask for help. Consequently, the program was not shown to be as effective on RAS as it was on RSES and HHI, which were more aligned with program objectives. The pilot study of an existing program — “The Power of Two” — was evaluated with limited participants (N = 7). The number of participants in this study was 45 and the study showed statistically significant changes with respect to recovery.

Among the originally developed items for process evaluation, only “I am able to communicate well with others about myself” and “I am able to listen to others well” regarding communication skills showed significant improvements. In interviews one month later, eleven participants said their overall communication had improved. Communication skills themselves are not a component of recovery but are essential for connecting with others [21]. For people who want to find love or get married, communication skills are crucial for achieving these goals. As mentioned in the interviews, this can be attributed to the fact that the role-playing exercises and the program itself were conducted in small groups, which provided ample opportunities to talk.

### Feasibility of the program

In terms of program satisfaction and usefulness in life, the positive ratings were more than 95%. Therefore, we believe that this program meets the needs of the participants, although some of the participants experienced negative consequences. Two participants had an unscheduled psychiatric visit, and seven people reported being negatively affected, but they did not have an unscheduled psychiatric visit. Of these nine participants, seven experienced heightened psychological emotions due to the program. The reasons for the negative effects were as follows: the interactions with others made them feel less confident; listening to others reminded them of a painful past memory; a person was hurt by the words of a program facilitator.

The program participants included people in a variety of situations: married, unmarried, and divorced. The program included content on parent-child relationship. Therefore, those who had not been successful in past marriages or who did not have a good relationship with their parents recalled unpleasant past events or compared themselves with others’ successes and lost confidence. Others felt stressed by people whose situations differed from their own. Many participants in the program described a positive change as they gained diverse ideas by sharing their thoughts and experiences with others, but others found it hard to compare themselves to others because of the diversity of program participants’ situations. Connectedness with others is an important part of recovery [3–5]; however, it can also diminish a person’s confidence. Social comparison theory [44] indicates that people evaluate themselves in comparison with others, but whether it will inspire them to achieve more, demoralize them by showing what they do not have, or make them feel incapable of achieving their goals is unknown. The previous reported program “The Power of Two” for developing romantic relationships was limited to single men aged 18–30. On the other hand, this program was diverse in terms of gender, diagnoses, and marital status; therefore, the group was less homogeneous and may have had less cohesion. In Japan, people with mental disabilities often join the same self-help groups and rehabilitation facilities, regardless of their diagnoses. Therefore, when considering the target population for this program, we believe that it is undesirable to limit the program based on diagnoses because some people will feel discriminated against by this exclusion. To achieve greater homogeneity of participants and the presence of married people in the group, it would be practical to only have unmarried participants in the program and to include married people as facilitators.

Furthermore, the competence of the program facilitator may be related to participants’ negative experiences. The program’s structure of being a group focused on people with mental disorders and facilitated by peer facilitators was mentioned as a positive aspect of the program by many participants. Therefore, training must be strengthened to increase the capacity of peer facilitators. Additionally, trained professionals who support peer facilitators appear to be important element.

Four of the applicants in this study were assessed by their psychiatrists to be unable to participate because of unstable psychiatric symptoms. It is undesirable for this program, which aims at recovery, to have individuals not deciding for themselves whether to participate in the program. However, some participants reported negative effects due to the program, although all recovered after one month. After this study was completed, we discussed with peer facilitators about whether we should continue to seek a psychiatrist’s permission to participate in the program. Many peer facilitators were concerned about the risk of worsening mental health conditions. Therefore, it was the peer facilitators’ decision to continue to encourage potential participants to consult a psychiatrist about program participation.

### Study limitations and future research

The first limitation of this study is that it was not an RCT, but a one-group pretest-posttest design. Therefore, we cannot rule out the possibility of other factors affecting the changes in outcomes. Second, the program facilitators received two days of training to conduct the program; however, most of them did not have professional qualifications (such as social work degrees). Therefore, they are not as well-trained in the successful use of group dynamics as those with professional education and training. Third, participants had multiple diagnoses. Peer-led groups are particularly useful in contexts in which people can relate to their peers. Therefore, this method may be less effective than one where participants have a single diagnosis. Fourth, we recognize that originally developed items may not have good validity or reliability. Since this pilot study does suggest the importance of communication and listening skills, future research needs to measure these constructs with psychometrically sound scales. Finally, diagnoses were self-reported rather than clinically assessed. Therefore, a valid diagnosis was not confirmed.

For future research, we believe that the program needs to focus on unmarried participants and to make use of peer facilitators (including those who are currently married) and to enhance the training of facilitators as well as provide professional support in conducting the program.

## Conclusion

This peer-led learning program on intimate and romantic relationships was effective to a moderate extent in improving recovery, especially in increasing self-esteem and enhancing hope. Two participants had unscheduled psychiatric visits that may have been due to their participation in the program, but all recovered after one month, so we consider the program feasible. For future research, the program needs modifications regarding the inclusion criteria of participants and the training of peer facilitators.

### Electronic supplementary material

Below is the link to the electronic supplementary material.

Additional file 1: Table S1. Negative changes up to one month after the program

## Data Availability

Access to the rules for participation and facilitation of this program was made openly available (https://kageyamaresearch.wixsite.com/airiki). The originally-developed simple AIRIKI guidebook is available upon request from the first author. The datasets generated and analyzed during the current study are not publicly available because of confidentiality concerns but are available from the corresponding author upon reasonable request after the approval of the institutional ethics review committee.
