# Supplementary material for: A peer-led learning program about intimate and romantic relationships for persons with mental disorders (AIRIKI): co-creation pilot feasibility study
Source: BMC Psychiatry. 2023 Oct 19;23:767. doi: 10.1186/s12888-023-05254-1 (PMC10588039; doi:10.1186/s12888-023-05254-1)
Supplement: Supplementary file 1 — Additional file 1: Table S1. Negative changes up to one month after the program [file 12888_2023_5254_MOESM1_ESM.docx]

| Table S1. Negative changes up to one month after the program | | |  |
| --- | --- | --- | --- |
|  |  | Reasons | Psychological burden due to program |
| Unscheduled psychiatric visits during first month after the program (n=2) | | | |
|  |  | Stressed during program because my situation was different from others | ✓ |
|  |  | Enjoyed program but participant’s mood become higher than normal | ✓ |
| No unscheduled psychiatric visits during first month after program (n=7) | | | |
|  | Dropout (n=3) | |  |
|  |  | Remembered painful experiences from program | ✓ |
|  |  | Hurt by program facilitator | ✓ |
|  |  | Physical condition |  |
|  | Unstable mental health condition (n=2) | |  |
|  |  | Seasonal instability of medical condition |  |
|  |  | Stressed as result of actions taken by attending program and because participant could not overcome challenges | ✓ |
|  | Less self-confidence (n=2) | |  |
|  |  | Others better communicators | ✓ |
|  |  | Others had successful marriages | ✓ |

Additional file 1
